# Speech outcomes in cochlear implant users depend on visual cross-modal cortical activity measured before or after implantation

**DOI:** 10.1093/braincomms/fcaf071

**Published:** 2025-02-14

**Authors:** Brandon T Paul, Vincent Trinh, Joseph Chen, Trung Le, Vincent Lin, Andrew Dimitrijevic

**Affiliations:** Department of Psychology, Toronto Metropolitan University, Toronto, ON M5B 2K3, Canada; Otolaryngology—Head and Neck Surgery, Sunnybrook Health Sciences Centre, Toronto, ON M4N 3M5, Canada; Otolaryngology—Head and Neck Surgery, Sunnybrook Health Sciences Centre, Toronto, ON M4N 3M5, Canada; Faculty of Medicine, Otolaryngology—Head and Neck Surgery, University of Toronto, Toronto, ON M5S 1A1, Canada; Otolaryngology—Head and Neck Surgery, Sunnybrook Health Sciences Centre, Toronto, ON M4N 3M5, Canada; Faculty of Medicine, Otolaryngology—Head and Neck Surgery, University of Toronto, Toronto, ON M5S 1A1, Canada; Otolaryngology—Head and Neck Surgery, Sunnybrook Health Sciences Centre, Toronto, ON M4N 3M5, Canada; Faculty of Medicine, Otolaryngology—Head and Neck Surgery, University of Toronto, Toronto, ON M5S 1A1, Canada; Otolaryngology—Head and Neck Surgery, Sunnybrook Health Sciences Centre, Toronto, ON M4N 3M5, Canada; Faculty of Medicine, Otolaryngology—Head and Neck Surgery, University of Toronto, Toronto, ON M5S 1A1, Canada; Evaluative Clinical Sciences Platform, Sunnybrook Research Institute, Toronto, ON M4N 3M5, Canada

**Keywords:** cochlear implants, speech outcomes, electroencephalography, cross-modal plasticity, alpha oscillations

## Abstract

Cochlear implants can partially restore hearing function in deaf individuals, but long-term speech listening outcomes vary widely across cochlear implant users. Visual cross-modal plasticity, where auditory cortical neurons upregulate visual inputs to assist visual processing, is one factor proposed to worsen cochlear implant users’ speech outcomes because it may limit auditory processing capability. However, evidence for this view is conflicting, and the relationship of cross-modal activity to speech perception may depend on other variables such as the type of visual activity and when it is assessed. To clarify, we measured visual cross-modal activity during a silent lip reading task using EEG in a cross-sectional, observational study. The study tested visual brain activation in 14 individuals prior to receiving a cochlear implant, 15 individuals tested at least 1 year after receiving and using a cochlear implant and 13 typical hearing controls who did not use a cochlear implant or hearing aid. Cross-modal responses to the onset of a visual event were positively correlated to speech outcomes in cochlear implant users tested after surgery but were negatively correlated in those tested prior to cochlear implant surgery. In addition, cross-modal increases in neural oscillatory power in the alpha band (8–12 Hz) arising in the lip reading task were associated with worse speech outcomes in both cochlear implant user groups. Taken together, results redress claims that cross-modal plasticity is maladaptive for speech outcomes and instead suggest that this relationship depends on the time point of testing, stage of sensory processing and likely the relevance of the stimulus for speech. In addition, findings form the basis for new neural markers that are predictive of cochlear implant users’ long-term speech ability.

## Teaser

Paul *et al*. report that ‘cross-modal’ visual brain responses measured from auditory cortex correlate to speech perception scores in cochlear implant users. However, the direction of the correlation depends on whether testing was done before or after cochlear implant surgery and on the stage of visual processing.

## Graphical Abstract

**Graphical Abstract:**
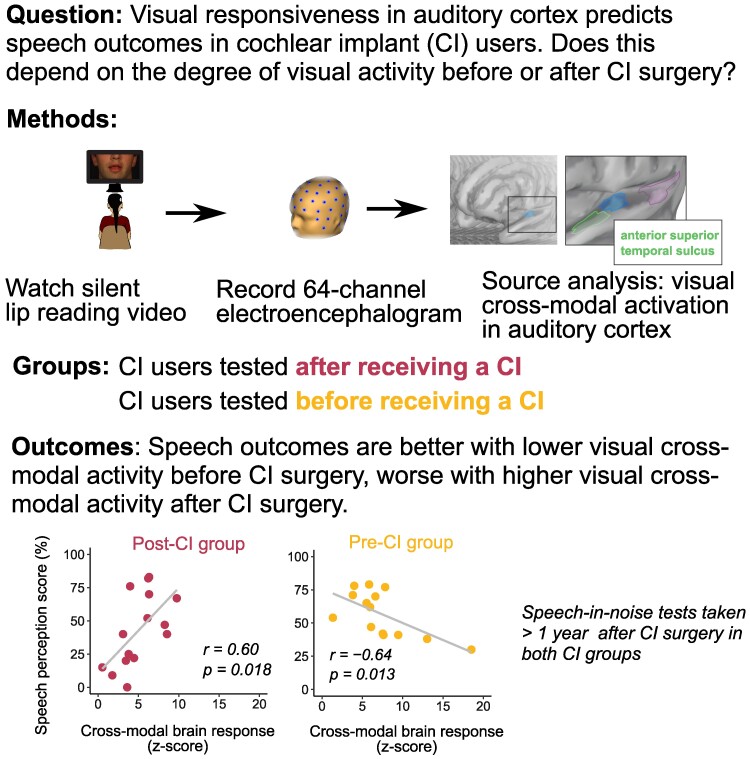

## Introduction

Cochlear implants (CIs) are surgically implanted devices that can partially restore hearing function in individuals with deafness or profound hearing loss. CIs work by electrically stimulating auditory nerve fibres using an electrode array inserted directly into cochlea. The electrical stimulation consists of current pulses shaped to convey features of speech and other sounds that are picked up by microphones on the CI earpiece. When CI users are first presented with speech after surgery, it is commonly unintelligible. However, through training and rehabilitation CI users learn to (re)associate this input with linguistic meaning, and speech perception becomes functional usually within several months to a year.^1,2^ However, the success of long-term speech perception varies widely. Many individuals exhibit excellent speech perception while others experience substantial difficulty even in ideal conditions. Some factors, such as the duration and cause of deafness and the age at deafness onset, are known to correlate with speech outcomes and may provide some prognostic value for speech ability.^3-5^ However, much of the inter-individual variability in CI users’ long-term speech outcomes remains unexplained.

One factor driving CI users’ speech outcomes is neural plasticity.^6^ For example, longer durations of CI use are linked to more extensive recruitment of cortical areas for auditory processing,^7,8^ which reflect neuroplastic changes that support speech recovery. However, neural plasticity in other senses, such as vision or touch, unfolds in response to deafness and changes with sensory experience. This plasticity can be intra-modal where cortical processing of an intact sense is improved within its respective sensory cortex. For example, visual cortical neurons become more responsive to visual input in deaf individuals.^8,9^ Cross-modal plasticity is where neurons in the deprived sensory cortex increasingly respond to intact senses. For instance, deafferented cortical neurons may upregulate latent cross-sensory inputs that are typically masked by intramodal activity.^10^ In deafness, auditory cortical neurons may shift to preferentially respond to visual or tactile input since auditory input is absent^11,12^ or reduced.^13^ Cross-modal and intra-modal plasticity are causally implicated in the development of adaptive behavioural changes that compensate for an organism’s sensory loss.^14^ For instance, deaf individuals show superior visual peripheral motion detection compared to hearing controls.^14,15^

Cross-modal plasticity occurs in human CI users whether deafness happens pre-lingually before language acquisition^16^ or post-lingually^17^ and is often revealed by measuring activation of auditory cortical areas following visual stimulation, comparing CI users to controls.^18^ Importantly, cross-modal plasticity has been consistently linked to CI users’ speech outcomes. Many studies argue that cross-modal plasticity has detrimental consequences on speech perception, for example, if auditory cortical neurons have downregulated their computational capacity for auditory processing and upregulate inputs from intact senses like vision. The evidence basis for this claim consists mainly of cross-sectional, observational studies in children and adults that correlate CI users’ speech perception scores to neural activity recorded from the auditory cortex or temporal brain regions, measured either before or after implantation. For example, children with higher resting-state metabolism in auditory cortex before implantation (taken as evidence of cross-modal involvement) show poorer speech scores after implantation,^19,20^ and CI users with poorer speech scores also show increased metabolic activity to visual stimulation when measured after CI surgery.^21^ In EEG studies, CI users with lower speech perception scores exhibit visual responses over temporal regions that suggest cross-modal recruitment.^9,22-25^ In contrast, better speech outcomes appear more likely when visual processing upregulates within visual cortical areas via intra-modal plasticity.^8,9,21^

Multiple lines of evidence, however, dispute the claim that cross-modal recruitment of auditory regions produces poor speech outcomes in CI users. In one animal study, cross-modal plasticity did not interfere with auditory cortical responsiveness to CIs^26^ and therefore was argued to not affect speech processing. In humans, Anderson *et al*.^27^ used functional near-infrared spectroscopy (fNIRS) to compare cross-modal activity evoked by visual speech in the superior temporal cortex of CI users before and after implantation. CI users whose cross-modal activity increased during this time had better speech scores, suggesting that cross-modal plasticity was beneficial. Interestingly, CI users in this study with stronger cross-modal activation to visual speech also had stronger activation of auditory cortex by auditory speech, implying that visual and auditory plasticity may cooperate.^27,28^ Zhou *et al*.^29^ report similar findings using fNIRS in children with CIs. Other studies show no relationship between cross-modal activity and speech perception ability in adult CI users.^21,30^ Reasons for the disagreement between studies remain unclear due to variance in stimulation and recording paradigms (evoked versus resting state), research designs (pre- or post-activity) and sample characteristics (pre-lingual or post-lingual deafness^16^). If these discrepancies were resolved, clinicians could be better equipped with neural makers that anticipate CI users’ speech listening difficulties, which further could inform new rehabilitation strategies that encourage optimal cooperation between visual and auditory modalities for speech ability.^28,31^

We previously used EEG to measure cross-modal responses in CI users in a lip reading task that involved a silent video clip of a face speaking a monosyllabic word.^32^ This active visual language task tapped into speech networks and differed from prior studies that are usually task-free and use low-level visual stimuli such as inverting chequerboards or apparent motion.^9,23^ We found that cross-modal activation to the onset of the video clip was positively correlated to CI users’ speech outcomes, which argues against the view that cross-modal plasticity is maladaptive for speech perception. However, in the same CI users, we found that cross-modal modulation of alpha (8–12 Hz) oscillations in auditory cortex during the lip reading task were negatively correlated with speech outcomes. No differences in intra-modal activation were found between CI users and controls. Our results suggested that the relationship between cross-modal activation and speech is more complex and depends on the stage of visual stimulus processing.

A limitation of our first study was that visual responses were measured in CI users who had been using their device for at least 1 year. Therefore, it was unclear if cross-modal plasticity prior to CI use would predict similar outcomes. In a new, separate analysis, we extended our methods to include a group of individuals tested prior to CI surgery, whose speech outcomes were measured 1 year after CI use. We used a new source analysis approach to test for differences in cross-modal activity across posterior and anterior regions of temporal cortex, since visual cross-modal plasticity may unfold outside of primary auditory cortex.^14,26,33^ We hypothesized that CI users tested pre-operatively (herein, the pre-CI group) will show similar relationships of cross-modal activity and speech-in-noise scores as CI users tested post-operatively (post-CI group): cross-modal brain responses to the onset of the video clip in both CI user groups will be larger compared to controls and will be positively correlated to speech-in-noise scores. The power of alpha rhythms during observation of lip reading will show individual differences where poorer speech perception will occur with increases in alpha power. Overall, results from our design will be the first to show how CI users’ speech outcomes are shaped by different stages of visual processing and the status of auditory afferentation (pre- or post-CI surgery).

## Materials and methods

### Participants

The study paradigm was first described in Paul *et al*.^32^ All CI users were recruited from the Sunnybrook Health Sciences Cochlear Implant Program. Demographic and hearing-related variables for the pre-CI group are presented in Supplementary Table 1. The pre-CI group consisted of 14 adults aged 32–74 (*M* = 56, SD = 16) with 8 females and 6 males. All but one pre-CI individual were post-lingually deafened. Participants in this group experienced deafness between 11 and 50 years (*M* = 26, SD = 14) before implantation. EEG measurements for visual cortical activity were completed in pre-CI participants within 12 months prior to implantation, on average of 5.6 months. Speech testing for those in the pre-CI group occurred 1 year after CI activation. Of these individuals, 6 were bilateral CI users at the time of speech testing but underwent EEG measurement prior to their first CI surgery. Eight individuals in this group used a CI with a hearing aid on the opposite ear. Two individuals used only a single CI. The post-CI group included 15 individuals (*M* = 57 years, SD = 20; 5 females, 10 males; Supplementary Table 2) with at least 1 year of CI experience, originally reported in Paul *et al*.^32^ All but four CI users were post-lingually deafened and had been using their CI between 1 and 20 years. The duration of deafness in this group ranged 9–62 years (*M* = 31, SD = 17). Participants in the pre-CI group were tested before receiving their first CI, if they were bilateral users. Participants in the post-CI group who were bilateral users were tested after receiving both CIs. Thirteen normal-hearing (NH) controls who were age-matched to the other groups (*M* = 55 years, SD = 21) also participated in the study. Therefore, our total sample size was 42. The sample size was determined by the availability of patients in the clinic who agreed to participate. No participants in any group reported use of sign language. Participants provided written informed consent and the study procedures aligned with the Research Ethics Board (REB) at Sunnybrook Health Sciences Centre (#474–2016) and accorded with the Declaration of Helsinki. Participants were compensated with money for their participation and were provided full reimbursement for parking at the hospital campus.

We measured speech-in-noise (SIN) ability in the pre- and post-CI groups using the Arizona Biomedical Institute (AzBio) sentence list. Stimuli were presented at 65 dBA in a sound booth through a single loudspeaker placed 1.5 m directly in front of each participant. Sentences were presented in speech-shaped noise at 60 dBA, yielding a + 5 dB signal-to-noise ratio. Performance was calculated as the percentage of correctly reported words across 50 sentences. Each participant was tested under their ‘best aided’ condition, which meant that tests were conducted with use of all devices, whether unilateral CI, bilateral CI, or bi-modal CI and hearing aid. Controls did not participate in AzBio testing because they typically perform at ceiling. CI users who were tested post-operatively underwent typical monitoring and rehabilitation at Sunnybrook Health Sciences Centre. Participants typically see the CI audiologist in our programme every 6 months. At these appointments, audiologists adjust programming as participants listen to audiobooks. No participant, either in the pre-CI or post-CI group, had a history of lip reading training. CI users removed their device prior to the lip reading task and EEG recording, described below. This was to prevent CI artefact from contaminating the EEG recording.

### Lip Reading task

**Figure 1:**
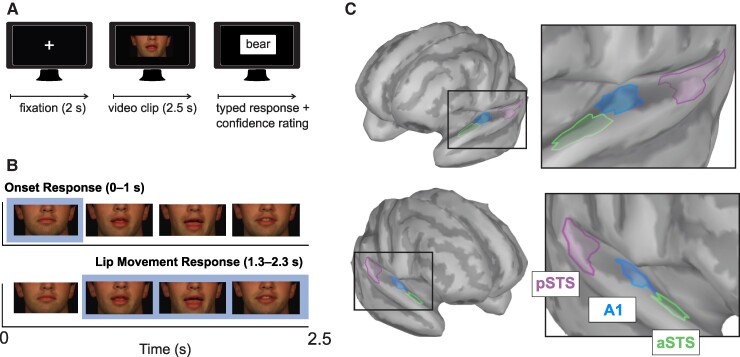
**Task design and cortical ROIs.** (**A**) Sequence of events in the lip reading task. On each trial, a fixation cross was shown for 2 s, followed by a silent video of a speaker articulating a single-syllable word for 2.5 s. One to two seconds after video offset, a textbox appeared where participants typed the word they perceived, and then rated the confidence in their answer. (**B**) Onset responses were analyzed in a one-second window after video onset, while the lip movement response was analyzed from 1.3 to 2.3 s after video onset. (**C**) ROIs were used to examine cross-modal responses. ROIs consisted of aSTS and pSTS, as well as the primary auditory cortex (A1). ROIs were examined in left and right hemispheres. aSTS, anterior superior temporal sulcus; pSTS, posterior superior temporal sulcus.

Stimuli in this study were a set of 2.5-s silent video clips of one male articulating a monosyllabic word used in our past study^32^ and in previous work.^34^ Only the nose, lips, and chin of the speaker were visible in the video clip. These clips were presented to participants on a visual display monitor 1 metre in front of participants. Participants viewed video clips in a lip reading task that required participants to type the word they perceived, shown in Fig. 1A. On each trial, participants were readied by presentation of a 2-s fixation cross on the computer screen, followed by the video clip. After the video ended, a fillable textbox appeared on the screen and participants typed the word into the textbox using a computer keyboard. One of the experimenters manually scored participant responses against the answer key and logged performance as the per cent of correctly identified words. In addition, participants were asked to rank their confidence in the response on a scale of 1 (‘not confident at all’) to 10 (‘absolutely confident’) after each trial, and these confidence ratings were averaged across all trials. Two hundred total video clips were presented in 10 blocks of 20 trials. Participants had the opportunity to take a break after each block. Before the lip-reading task, participants received a list of 46 monosyllabic words and were told that these stimuli may appear in the videos. They were allowed to study the list for 2 min. Not all words were presented to participants.

### EEG recording and processing

For the duration of the lip reading task, we recorded the 64-channel EEG at a rate of 2000Hz through a Neuroscan amplifier (Compumedics, Victoria, Australia) adapted for an actiChamp Brain Products cap (Brian Products GmbH, Inc., Munich, Germany). The electrode montage used an equidistant electrode placement as opposed to a 10–20 system to facilitate source localization as reported in our previous studies.^32,35^ In this setup, the reference channel is at the vertex (sensor Cz) and the ground electrode 50% of the distance to the nasion. The spatial layout of the electrodes was digitized into a 3-dimensional format using a Polhemus FastTrack digitizer (Colchester, USA).

In Brain Vision Analyzer software v2.0 (Brain Products GmbH Inc., Munich, Germany), a.01 Hz high-pass filter first removed baseline drift. EEG data were then downsampled to 500 Hz, and movement artefacts >500 mV were visually identified and removed. Independent components analysis (ICA) was used to identify and suppress spatiotemporal patterns of ocular, cardiac, and transient electrode artefacts. Continuous data were then exported to MATLAB for analysis with the Brainstorm toolbox.^36^

We examined two types of cross-modal activity, onset responses to the first appearance of each video clip and sustained neural oscillations during the period in the video where lips were moving to articulate the word (Fig. 1B). Source analysis of visual onset responses was performed using standardized low-resolution electromagnetic tomography (sLORETA) modelling^37,38^ in the Brainstorm toolbox in MATLAB using default settings. First, we calculated the visual evoked potential by re-referencing the data to the common average, and epoching each trial from −0.25 s before video onset to 1 s after onset. After sLORETA modelling, the absolute value of the model was taken followed by *z*-score baseline normalization in Brainstorm, which limits the bias in source estimation for superficial generators and permits comparison across regions and groups. We used the OpenMEEG plugin in brainstorm to create boundary element model (BEM) head models, and each sLORETA map was used to extract the activation time series from 0 to 1 s after video onset, as shown in Fig. 1B, top row. Time series were extracted in predefined regions of interest (ROIs) based on the Desikan–Killiany atlas,^39^ following regions suggested by Stropahl and Debener^40^ and as we have done in past studies on VEPs and CI users.^30,32^ The primary auditory ROI (herein, A1) was selected to approximate Brodmann areas 41 and 42. Two additional ROIs (per hemisphere) were chosen, anterior superior temporal sulcus (aSTS) and posterior superior temporal sulcus (pSTS). All three ROIs had similar surface areas. Group comparisons were performed on the ROIs’ time-waveform activation on a 20-ms time window centred on the N1 peak amplitude. ROIs are shown on head maps in Fig. 1C.

We used time–frequency analysis to examine the power of neural oscillations during the lip reading period. Continuous EEG data were first referenced to the common average, epoched from −1 to 5 s relative to the onset of the video and subjected to time-frequency analysis in Brainstorm. Morlet wavelets were used with a 1 Hz central frequency and full width half maximum at 3 s, and power was computed in 1 Hz steps from 1 to 40 Hz. After, power values normalized as event-related synchronization/desynchronization (ERS/ERD), calculated by subtracting the mean baseline power.^41^ We used the LCMV beamformer in Brainstorm for source reconstruction using default settings. To extract alpha power, we averaged power values from 8 to 12 Hz from the period of 1.3 s to 2.3 s, which represents the period in each video clip where lip movement was occurring. The same ROIs used for imaging the N1 onset response were used for the LCMV beamformer.

### Statistical analysis

Statistical analyses were performed in the R statistics package.^42^ Behavioural performance and demographic variables were analyzed between groups using unpaired Welch’s t-tests. Cross-modal activity in source space was compared between all 3 groups (pre-CI, post-CI and Normal Hearing) within each ROI using mixed analysis of variance (ANOVA; *afex* package in R). Degrees of Freedom were adjusted with Greenhouse Geisser correction for a lack of sphericity in ANOVA models. The *emmeans* package was used to conduct post-hoc tests for each ANOVA model, which were corrected for false discovery rate (FDR). We used Pearson correlations to examine the strength of association between behavioural variables, speech-in-noise scores as speech outcomes, onset responses in each ROI, and induced alpha power in each ROI. For all tests, the alpha criterion for Type I error was.05, and all tests were two-tailed. ANOVA models are reported with generalized eta squared (η²) values to express effect size. Results plots were made using Brainstorm in MATLAB and the *ggplot2* package in R.

## Results

### Behavioural results

Across all groups, the average performance on the lip reading task was 27.8% (SD = 13.2%). Descriptively, the post-CI group had the highest scores (*M* = 31.7%, SD = 16.0%) compared with the pre-CI group (*M* = 26.4%, SD = 11.5%) and NH group (*M* = 25.0%, SD = 11.2%). However, groups did not significantly differ (*P* > 0.21). Self-rated confidence in task performance was the highest in the post-CI group (*M* = 6.0 out of 10, SD =1.9), which was significantly higher than the pre-CI group [*M* = 3.9, SD = 2.1; *t*(26.27) = 2.69, *P* = 0.012], but the NH group (*M* = 5.1, SD = 1.8) did not differ from either CI group (*P* > 0.14). Within each group, we explored correlations between confidence ratings and task performance. Lip reading performance in the post-CI group was positively associated with confidence ratings (*r* = 0.67, *P* = 0.009), but similar trends were not significant in the pre-CI (*r* = 0.51, *P* = 0.052) or NH groups (*r* = 0.45, *P* = 0.12). In sum, lip-reading behaviours were not different between groups although the post-CI group had higher confidence than the pre-CI group. There was a tendency within each group for better performance with higher confidence ratings, although this was only significant for the post-CI group.

Speech-in-noise scores (AzBio Sentences at +5 dB SNR) measured at least 1 year after CI activation were descriptively higher in the pre-CI group (*M* = 57%, SD = 16.9%) than the post-CI group, which had wider variability (*M* = 43%, SD = 27.7%); however, group performance did not significantly differ (*P* = 0.12). Speech-in-noise scores were not correlated to lip-reading performance or confidence ratings in either CI group (*P* > 0.11).

### Cortical onset responses

**Figure 2:**
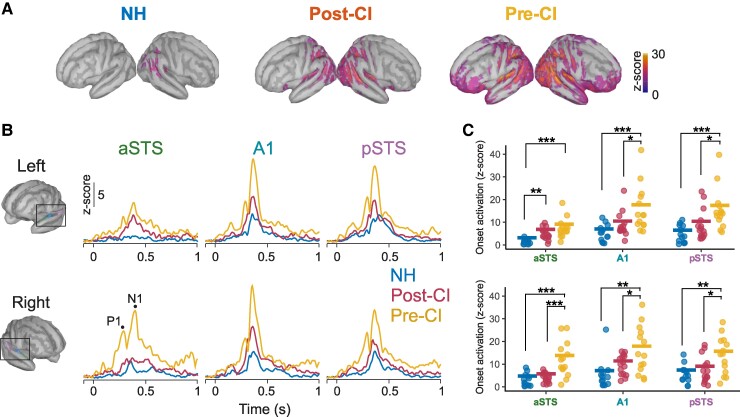
**Cross-modal activation to the onset of the video clip**. (**A**) Whole-brain sources of cross-modal onset activation for NH, post-CI and pre-CI groups. Shading shows strength of activation as a *z*-score, with higher *z*-scores indicating stronger responses. Shading is thresholded at a *z*-score of 7.5, 25% of the maximum value of the grand average). (**B**) Time courses of onset responses as *z*-scores for each group’s grand average. The top row shows responses from the left ROIs, and right ROIs are plotted on the bottom row. (**C**) Onset activation shown for each individual (dots) in each group. The top row shows left hemisphere ROIs and the right-sided ROIs are on the bottom row. Thick horizontal lines show the group average. Asterisks denote significant group differences. (**C**) Individual data points represent one CI or NH participant. *N* = 15 in the post-CI group, *N* = 14 in the pre-CI group, and *N* = 13 in the NH group.*P < 0.05, **P < 0.01, ***P < 0.001. Significance levels reflect results from FDR-corrected post-hoc tests that followed ANOVA models. A1, primary auditory cortex; aSTS, anterior superior temporal sulcus; NH, normal hearing; pSTS, posterior superior temporal sulcus.

Cross-modal activation to the onset of the movie for each group and auditory ROI is shown in Fig. 2. Figure 2A shows whole-brain sources for onset responses at the peak N1 response. Note that responses in both CI groups show strong recruitment of auditory cortical regions compared with NH controls. Time courses of onset activation are shown in Fig. 2B for each ROI. Descriptively, those in the pre-CI group showed the strongest responses, followed by the post-CI group, and NH controls had weaker responses. Clear N1 responses are shown in all groups except for N1 responses in the left aSTS in the NH group, which did not exhibit a clear onset morphology. Note that P1 responses were apparent in the pre-CI group but were weaker or absent in the post-CI and NH groups and therefore P1 responses were not analyzed.

Figure 2C shows N1 onset responses at the individual level, compared across hemisphere, ROI, and group. A 3 × 3 × 2 mixed ANOVA modelled onset activation as a function of group (between-subjects factor), hemisphere (left versus right; within-subject) and ROI (anterior (a)STS, posterior (*P*)STS, and A1; within-subjects). The model returned main effects of group [*F*(2,39) = 15.61, *P* < 0.001, η^2^ = 0.298] and ROI [*F*(1.58, 61.77) = 21.43, *P* < 0.001, η^2^ = 0.108], and a three-way interaction between group, ROI, and hemisphere [*F*(3.77, 73.56) = 3.03, *P* = 0.025, η^2^ = 0.014]. *Post hoc* tests with FDR correction indicated that both CI groups had stronger activation magnitudes compared with NH controls in left aSTS (*P* < 0.005), suggesting evidence of increased cross-modal responses. Pre-CI and post-CI groups did not differ in this region (*P* = 0.059). However, for all other ROIs, the pre-CI group had larger activation magnitudes than post-CI users and NH controls (*P* < 0.036), and no other differences were found between the CI and NH groups (*P* > 0.16).

### Alpha ERS/D during lip movement

**Figure 3:**
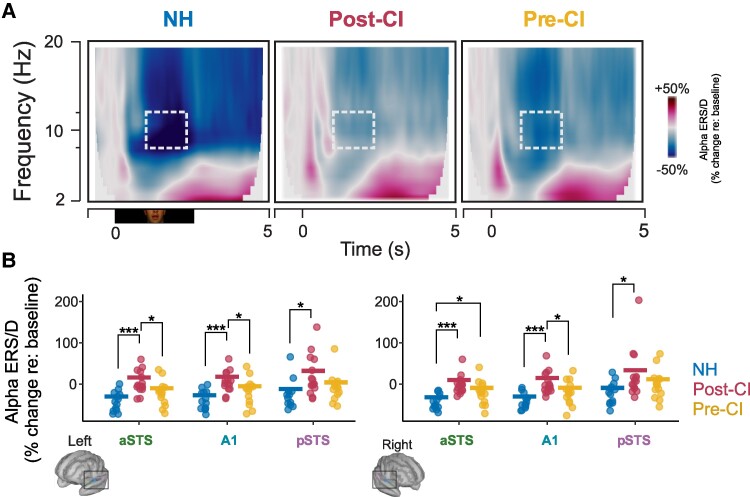
**Cross-modal modulation of alpha rhythms during observation of lip movement.** (**A**) Time-frequency plots showing increases (ERS) and decreases (ERD) of oscillatory power relative to baseline, as a function of time and frequency. The lip movement video lasted 2.5 s in duration, and a schematic of the stimulus period is plotted under the leftmost plot. White dashed boxes outline the time and frequency window that was averaged for each person to compute their alpha power. Each column represents one of the participant groups. (**B**) Alpha power shown for each individual (dots) in each group. The left panel shows left hemisphere ROIs and the right-sided ROIs are on the right panel. Thick horizontal lines show the group average. Asterisks denote significant group differences. (**B**) Individual data points represent one CI or NH participant. *N* = 15 in the post-CI group, *N* = 14 in the pre-CI group, and *N* = 13 in the NH group.*P < 0.05, **P < 0.01, ***P < 0.001. Significance levels reflect results from FDR-corrected *post hoc* tests that followed ANOVA models. A1, primary auditory cortex; aSTS, anterior superior temporal sulcus; NH, normal-hearing; pSTS, posterior superior temporal sulcus.

Induced alpha ERS/ERD across ROIs measured during lip reading and sourced to auditory ROIs is shown in Fig. 3. Figure 3A shows induced neural oscillations across 2–20 Hz frequency bands averaged over hemisphere and ROI, as a grand average for each group. These plots show modulations of oscillatory power across all frequency bands for the onset and duration of the lip reading video clip. These modulations include increases in power (ERS, event-related synchronization) and decreases in power (desynchronization, ERD). A clear ERS occurs at video onset, but after onset as lip movement is occurring, ERD occurs in the alpha band, which seems to differ across groups. A 3 × 3 × 2 mixed ANOVA compared this response across groups using the same factors as reported for onset activations. The model returned main effects of group [*F*(2,39) = 9.01, *P* < 0.001, η^2^ = 0.257] and ROI [*F*(1.05,41.05) = 22.80, *P* < 0.001, η^2^ = 0.072]. Interactions and the main effect of hemisphere were not significant (*P* > 0.063). Individual-level responses are shown for group, hemisphere, and ROI in Fig. 3B. FDR-corrected *post hoc* tests suggested that alpha ERS/ERD was higher in the post-CI group compared to NH controls at all ROIs (*P* < 0.032), and higher than the pre-CI group in bilateral A1 and aSTS (*P* < 0.047). While alpha ERS in the pre-CI group appeared larger than the NH group, this difference was only significant in the right aSTS (*P* = 0.031).

### Brain–behaviour correlations

**Figure 4:**
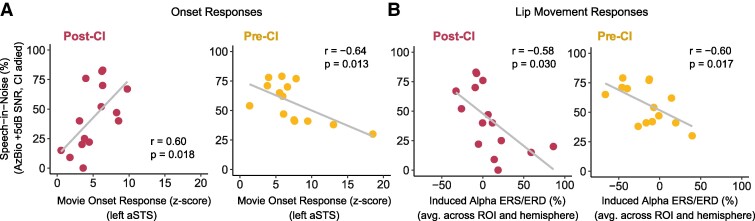
**Correlations between cross-modal neural activity and CI users’ speech outcomes**. Speech outcomes indicate performance on the AzBio sentence test, administered in speech-shaped noise at SNR of +5 dB while participants were CI-aided. (**A**) Pearson-product moment correlations between onset responses and speech outcomes. (**B**) Pearson-product moment Correlations between alpha power modulations and speech outcomes. In each plot, grey lines represent the least squares line. Correlations are shown with Pearson’s correlation coefficients and the associated *P*-values. (**A** and **B**) Individual data points represent one CI user. *N* = 15 in the post-CI group, *N* = 14 in the pre-CI group. aSTS, anterior superior temporal sulcus; CI, cochlear implant; ROI, region of interest.

In both CI user groups, we compared speech-in-noise scores to onset activation magnitudes in left aSTS because the CI groups showed significantly higher cross-modal activation compared with NH controls at this ROI. Shown in Fig. 4A, left panel, left aSTS activation at onset was positively correlated to speech-in-noise performance in the (*r* = 0.60, *P* = 0.018) for the post-CI group, but strikingly, left aSTS activation negatively correlated to speech-in-noise scores in the pre-CI group (*s* = −0.64, *P* = 0.013; Fig. 4A, right). Using Fisher’s r-to-z transformation, these correlation coefficients were significantly different (*z* = 3.477, *P* < 0.001). Thus, the relationship between cross-modal activation and speech-in-noise performance in CI users depended on when visual evoked brain activity measurements were taken. Exploratory analyses suggested that onset responses in other ROIs were not correlated with speech outcomes in either CI group (*P* > 0.1) except for the left primary ROI in the pre-CI group, which like left aSTS, was negatively correlated with speech-in-noise scores.

Similar correlations were examined between speech-in-noise scores and alpha ERS/D during observation of lip movement. Since group differences did not depend on hemisphere or ROI, we collapsed across these latter two factors before running correlations. Alpha ERS/D was negatively correlated to speech-in-noise scores for both the pre-CI group (*r* = −0.58, *P* = 0.030) and the CI group (*r* = −0.60, *P* = 0.017), indicating that worse speech scores were associated with alpha ERS (increases in alpha power relative to baseline) during lip movement observation.

Correlations between speech-in-noise performance and both onset responses and alpha ERS/D suggest the possibility that brain response types also correlate within CI groups. Participants in the post-CI group with higher alpha power had smaller onset responses (*r* = −0.59, *P* = 0.019), which were associated with lower speech-in-noise scores. While the correlation between alpha power and onset magnitude was expectedly positive for CI users tested pre-operatively (*r* = 0.45), this result was not significant (*P* = 0.10). Finally, we tested whether participant age, their duration of deafness, and age at deafness onset mediated relationships between cross-modal brain responses and speech-in-noise outcomes in both CI groups, using the *mediate* package in R. No significant mediating effects were found (*P* > 0.19).

We also explored correlations between lip reading behaviour (confidence and performance) and cross-modal brain activation (onset activation and alpha power). No significant correlations were found between measures in any group after *P*-values for FDR were implemented. When left uncorrected, the only significant positive correlation was found between lip reading performance and alpha power in the post-CI group (*r* = 0.56, uncorrected *P* = 0.03).

## Discussion

There is disagreement whether visual cross-modal recruitment of auditory cortex affects CI users’ long-term speech function. A resolution to this issue would help to optimize and predict CI users’ speech outcomes. We examined source-localized cross-modal responses in a group of individuals prior to CI surgery, a group > 1 year after surgery, and in normal-hearing age-matched controls. Cortical responses to the onset of the visual stimulus were larger in both CI groups, suggesting evidence cross-modal recruitment compared to controls. Cross-modal modulations of cortical alpha power related during observation of lip movement also differed between groups. Nearly all NH controls showed decreased alpha power during this time, but in both CI groups, participants exhibited both increases and decreases in alpha power. These cross-modal responses were also correlated with speech-in-noise outcomes; increased alpha power during lip movement occurred with worse speech-in-noise performance and decreased power was linked to better performance. Second, and in contrast with our hypotheses, correlations between speech-in-noise scores and visual onset responses differed between CI groups. Speech outcomes and cross-modal responses were positively correlated in the post-CI group and were negatively correlated in the pre-CI group. The results overall suggest that the link between speech outcomes and cross-modal brain activity in CI users depends on the type of stimulus processing and whether a person is using a CI at the time when visual brain activity is measured. These findings run counter to the narrative that cross-modal plasticity is uniformly maladaptive for CI users’ speech recovery.

Cross-modal activity was measured while participants engaged with a lip-reading task. We did not find evidence that CI users were better at the lip reading task compared to typical hearing controls, contradicting prior studies showing better performance in CI users (e.g.^17^). For further discussion, see Paul *et al*.^32^

### Cross-modal brain responses in CI users and controls

Larger cross-modal responses to the onset of the video clip in the CI groups are consistent with evidence of cross-modal recruitment associated with deafness or profound hearing loss and agree with many EEG studies showing elevated cross-modal responses in children^24^ and adults with CIs.^9,18,30,40^ Participants in this study who were tested before implantation had larger onset responses in all auditory ROIs compared to NH controls and CI users tested after implantation, suggesting that before implantation, auditory regions are strongly implicated in visual processing. The latter post-CI group presumably had smaller onset responses to the pre-CI group because auditory cortical regions had now re-engaged with auditory processing.^17^ Individuals in the post-CI group had larger responses to the onset of the video clip compared to controls but only in the left anterior temporal ROI, raising questions about the lateralization of cross-modal effects. The onset response was evoked by presenting the lower half of a face, which differs from past research that uses full-face images and shows right-lateralized effects.^18,40^ The full versus half-face difference may account for this disagreement in lateralization, but left-sided involvement should not be unexpected because the measurements were embedded in a visual speech task, which is known to activate left-lateralized auditory cortical areas,^43^ or more generally left-lateralized language networks;^44^ for further discussion, refer to Paul *et al*.^32^

Few studies have examined visual-evoked neural oscillations in human CI users or deaf individuals. Here, we show that CI users exhibit variability and show increases or decreases in alpha power to the same stimulus. Reduced alpha power to visual events in parietal areas has been shown in CI users with orthographic characters^30^ and in early deaf individuals with moving circle stimuli.^45^ However, wide differences in visual stimulation paradigms and group characteristics limit conclusions that can be drawn from these few studies. More generally, alpha oscillations measured by EEG have been hypothesized to reflect active inhibitory processes through cortical inhibitory interneurons.^46-48^ When applied to the present data, the inhibition framework may suggest higher alpha power reflects visually driven inhibition to temporal regions in some CI users, while lower power represents the inverse. This assumption requires further testing. Interestingly, alpha power was overall highest in the post-CI group, raising the potential that this neural activity is altered following re-afferentation of the auditory system.

### Speech outcomes and cross-modal recruitment of auditory cortex

Brain plasticity is a determinant of speech recovery in CI users,^3^ and intra-modal plasticity in the visual system is viewed as beneficial for speech outcomes because it promotes auditory-visual interactions that facilitate speech perception.^2,8,9,21^ However, when visual processes recruit auditory cortical regions, speech outcomes appear to be worse, and researchers speculate whether use of visual language such as lip reading or sign language drives this cross-modal effect.^9,19,20,22-25,49,50^ For this reason, cross-modal plasticity has been viewed as a maladaptive process for sensory restoration. Yet, other studies argue that visual cross-modal recruitment of auditory cortex is beneficial for auditory recovery,^26,27,29,51,52^ which may reflect activity that reinforces multimodal speech representations.^8,27^ In addition, visual language use may not be the driver of cross-modal plasticity.^53^ Our results may clarify these disagreements when contextualized in past research: (i) We show evidence that cross-modal activity was not consistently associated with worse speech outcomes. (ii) We showed that the stage or type of visual processing also differs in shaping speech outcomes. (iii) In light of past findings, we argue that the relationship between cross-modal plasticity and speech outcomes may depend on the relevance of the visual stimulus to language.

First, evidence for higher cross-modal recruitment in the onset responses was linked to poorer outcomes in individuals tested pre-operatively and better outcomes in CI users tested post-operatively. These contrasting correlations do not agree with the view that cross-modal plasticity necessarily explains worse speech performance. Alternatively, individuals who have less cross-modal recruitment before CI surgery and more recruitment after surgery may be effectively leveraging visual speech cues during rehabilitation and therefore show better overall speech functioning.^8,27^ CI users who have large cross-modal responses before CI surgery may be more reliant on visual cues to compensate for deafness but may try to suppress visual processing in favour of auditory processing in a way that could downregulate cross-modal recruitment. This could produce worse speech outcomes. A problem with this interpretation is that pre- and post-CI groups in this study consisted of different individuals. A longitudinal design would be preferred to track the trajectory of cross-modal activity before and after CI surgery to substantiate these claims.

Second, an important new finding was that CI users with worse speech outcomes, irrespective of group, had increased alpha power during observation of lip movement while CI users with better speech outcomes had lower power. Because this configuration of findings differed from the correlations between speech outcomes and cross-modal onset responses, we suggest that onset responses and sustained alpha oscillations reflect a distinct stage of visual stimulus processing. Furthermore, onset responses and alpha power were negatively correlated in the post-CI group but tended to be positively correlated in the pre-CI group, despite the latter correlation not reaching statistical significance. As stated, the role of visual cross-modal modulation of alpha oscillations is not immediately clear. If increased alpha oscillations represent functional inhibition,^46,47^ one interpretation is that cross-modal inhibition of auditory cortical areas is linked to worse speech outcomes and cross-modal excitation occurs with better speech outcomes. This agrees with the view that cross-modal activation is beneficial for speech recovery and matches findings from Anderson *et al*.^27^ and Zhou *et al*.^29^ showing better auditory speech scores in CI users with larger visual speech-evoked metabolic responses in temporal cortex. On the other hand, findings can be interpreted under the view that cross-modal recruitment is maladaptive. Increased inhibitory activity could accompany a general upregulation of visual inputs, meaning that higher alpha power reflects more cross-modal recruitment and therefore worse speech outcomes. However, because CI users tested post-operatively with better speech scores had stronger onset responses (evidence of more cross-modal recruitment), a maladaptive explanation is less likely.

Finally, cross-modal activation may differ depending on the relevance of the stimulus for language processing and may differentially predict separate speech outcomes. Although we did not test for cross-modal activation differences between speech and non-speech stimuli, comparison across the literature suggests that this difference matters for speech outcomes. Interestingly, studies showing positive relationships between speech perception and cross-modal activity tend to use visual speech,^27,29^ including the current post-CI data (but see Strelnikov *et al*.^21^). However, other studies have shown negative relationships between speech outcomes and cross-modal responses for non-speech stimuli such as inverting chequerboards, apparent motion, or other low-level stimuli.^9,22-25^ Indeed, studies that compare visual cross-modal responses between stimulus types suggest that visual speech-relevant stimuli create stronger activation compared to non-speech stimuli.^18,52^ Fullerton *et al*.^52^ compared speech outcomes to visual speech and non-speech activation in CI users using a functional connectivity analysis from fNIRS recordings. Stronger functional connectivity between left auditory regions and visual cortex were linked to better speech outcomes, providing further evidence for synergistic effects of speech-relevant audio-visual interactions in supporting CI users’ speech recovery.

One limitation of our study was that we did not collect any data on daily CI use. The duration of daily CI use has been shown to correlate to CI users’ speech outcomes.^54-56^ For example, participants in the post-CI group may have had better speech scores and stronger onset responses if they use their devices for comparatively longer durations each day. Future research should account for daily CI use, among other factors to strengthen claims about speech outcomes and cross-modal activity.

## Conclusions

We provide new evidence clarifying how cross-modal plasticity relates to speech outcomes in CI users. Importantly, cross-modal recruitment under some conditions may be linked to better speech outcomes, but more generally, this association may be dependent on the conditions in which it is measured. These conditions include time point of testing (pre- versus post-implantation), stage of visual processing (onset versus sustained responses), and perhaps whether the visual events reflect speech signals. We recommend that progress can be made with new studies that include longitudinal monitoring of cross-modal activity before and after CI surgery, which would also compare stimulus processing stages for speech and non-speech visual events. Cross-sectional studies, such as the present design and most previous work on this topic, limits conclusions because the evolution of cross-modal plasticity cannot be tracked. To our knowledge, only Anderson *et al*.^27^ and Rouger *et al*.^17^ used longitudinal designs, and neither study reports evidence that worse speech outcomes occur with increased cross-modal recruitment.

## Supplementary Material

fcaf071_Supplementary_Data

## Data Availability

Data files used in the analysis are available on the Open Science Framework (https://osf.io/xnjzg/).
