# Supplementary material for: Speech outcomes in cochlear implant users depend on visual cross-modal cortical activity measured before or after implantation
Source: Brain Commun. 2025 Feb 14;7(1):fcaf071. doi: 10.1093/braincomms/fcaf071 (PMC11851104; doi:10.1093/braincomms/fcaf071)
Supplement: fcaf071_Supplementary_Data [file fcaf071_supplementary_data.docx]

**Supplementary material**

**Supplemental Table 1: Post-CI Group Demographics**

| ID | Age | Sex | Deafness onset age | Deafness duration (yrs) | Deafness etiology | CI Side | Other device | CI use (yrs) | |  |  |
| --- | --- | --- | --- | --- | --- | --- | --- | --- | --- | --- | --- |
|  |  |  |  |  |  |  |  | Left | Right | AzBio + 5 dB SNR (%) |  |
| CI01 | 18 | M | Birth | 18 | Unknown | Bilateral | - | 3 | 15 | 83 |  |
| CI02 | 25 | F | Birth | 25 | Hereditary | Right | None | - | 20 | 40 |  |
| CI03 | 28 | F | 3 | 26 | Unknown | Right | None | - | 16 | 0 |  |
| CI04 | 54 | M | 9 | 45 | Suspected Hereditary | Right | None | - | 2 | 40 |  |
| CI05 | 54 | M | 22 | 32 | Suspected Hereditary | Right | Right HA | - | 2 | 67 |  |
| CI06 | 60 | F | Birth | 60 | Suspected Hereditary | Bilateral | - | 10 | 17 | 15 |  |
| CI07 | 62 | F | 15 | 47 | Unknown | Right | Left HA | - | 6 | 22 |  |
| CI08 | 62 | M | Birth | 62 | Hereditary | Left | Right HA | 13 | - | 20 |  |
| CI09 | 63 | M | 40 | 23 | Meniere's disease | Left | Right HA | 4 | - | 76 |  |
| CI10 | 63 | M | 16 | 47 | Bilateral Otosclerosis | Right | Left HA | - | 2 | 82 |  |
| CI11 | 71 | M | 56 | 15 | Unknown | Left | Right HA | 6 | - | 47 |  |
| CI12 | 72 | M | 61 | 12 | Unknown | Left | Right HA | 1 | - | 52 |  |
| CI13 | 74 | F | 48 | 26 | Suspected Hereditary | Right | Left HA | - | 4 | 70 |  |
| CI14 | 74 | M | 55 | 20 | Unknown | Left | Right HA | 11 | - | 9 |  |
| CI15 | 78 | M | 70 | 9 | Hereditary/Noise exposure | Left | None | 1 | - | 25 |  |

**Supplemental Table 2: Pre-CI Group Demographics**

| ID | Age | Sex | Deafness onset age | Deafness duration (yrs) | Deafness etiology | CI Side | Other device | Months between testing and CI activation | |  |  |
| --- | --- | --- | --- | --- | --- | --- | --- | --- | --- | --- | --- |
|  |  |  |  |  |  |  |  | Left | Right | AzBio + 5 dB SNR (%) |  |
| PCI01 | 32 | F | 1 | 31 | Hereditary | Right | Hearing aid | - | 5 | 78 |  |
| PCI02 | 32 | M | 17 | 15 | Unknown | Bilateral | - | 16 | 6 | 77 |  |
| PCI03 | 35 | F | 9 | 26 | Otosclerosis/Unknown | Bilateral | - | 5 | 40 | 62 |  |
| PCI04 | 37 | F | 14 | 23 | Unknown | Left | Hearing aid | 1 | - | 70 |  |
| PCI05 | 54 | F | 33 | 21 | Unknown | Right | None | - | 3 | 65 |  |
| PCI06 | 55 | M | 41 | 14 | Unknown | Right | Hearing aid | - | 6 | 41 |  |
| PCI07 | 58 | M | 9 | 49 | Hereditary | Bilateral | - | 34 | 8 | 30 |  |
| PCI08 | 62 | F | 38 | 24 | Unknown | Left | Hearing aid | 11 | - | 79 |  |
| PCI09 | 66 | F | 55 | 11 | Unknown | Bilateral | - | 6 | 30 | 54 |  |
| PCI10 | 66 | M | 16 | 50 | Noise Exposure | Left | Hearing aid | 8 | 22 | 41 |  |
| PCI11 | 72 | M | 54 | 18 | Unknown | Right | Hearing aid | - | 12 | 71 |  |
| PCI12 | 73 | F | 50 | 23 | Turner Syndrome | Left | Hearing aid | 7 | - | 42 |  |
| PCI13 | 74 | F | 23 | 51 | Unknown | Right | Hearing aid | - | 5 | 38 |  |
| PCI14 | 74 | M | 59 | 15 | Unknown | Right | None | - | 1 | 47 |  |
